# Supplementary material for: Flowing between gongs: Mixed-methods insights into shared flow and temporal distortion in music performance
Source: PLoS One. 2025 Feb 10;20(2):e0302769. doi: 10.1371/journal.pone.0302769 (PMC11809782; doi:10.1371/journal.pone.0302769)
Supplement: S1 File — Semi-structured interview protocol for the four focus groups. (DOCX) [file pone.0302769.s001.docx]

**S1 File. Focus group protocol.** Semi-structured interview protocol for the four focus groups.

***Thank you so much for participating my study today. This focus group will now investigate your perceptions of flow, and playing gamelan in general, as a group.***

- What was your experience of playing in today’s experiment?

*Did you play well, did it go as planned?*

*How would you compare the different pieces, and improvising?*

- What is your understanding of flow state?

*How might it be defined, and what might it involve?*

***Flow state defined primarily as a balance between challenge and skills for a given task and moderate mental effort, but also includes ideas of clear goals, absorption and more. This results in being optimally challenged, so that you aren’t bored but aren’t stressed. You might have also heard about this being referred to as ‘being in the zone’.***

- What was your experience of flow state while playing in this experiment?

*Do any of you think you might have felt this at any point today? When?
If not, why not?*

***Something I’m interested in is group flow. For some researchers, shared flow or group flow is often seen as an extension of individual flow state, where it typically arises through synchronous or coordinated action, and relates to the level of togetherness a group might feel.***

- - What was your experience of group flow state while playing in this experiment?

*Do any of you think you might have felt this at any point today? When?
If not, why not?*

- - What was your experience of the differences between individual flow state and group flow while playing in this experiment?

*Do you think they might occur in alternation? How might this come about?*

- - In general, what do you think about the relevance of group flow state to gamelan playing?

*How might it arise in gamelan playing?*

*Do you all agree? Does anyone have a different experience?*

- In general, do you think there’s a difference in the potential for group flow between improvised and traditional playing? If so, how would you describe this?

*Do you all agree? Does anyone have a different opinion?*

- Please describe an instance outside of this session in which you have experienced group flow state.

*How was it experienced?*

*Do you all agree?*

*Do you think you are more likely to enter a feeling of flow or group flow when you are playing a certain instrument or kind of piece?*

***One of the main focuses of this experiment is to isolate and assess one parameter of flow states, the perception of time.***

- In general, how do you perceive time when you play? Can you describe this as a group?

*Can you reach a consensus that everyone agrees upon?*

*Does anyone disagree? Feel the opposite? Can you describe this?*

*Did you feel this today, is this how you usually feel?*

- - How do you think time perception might relate to flow experiences?

*Do you think you might be more likely to be in flow if you perceive time as accurately, or faster or slower?*

- Is there anything about your experience of playing in this experiment that might have influenced any of these ideas we have discussed on time and flow?

*For instance, do you think the electrodes were distracting? Or do you think the experiment set-up might have interfered with the potential for flow to occur?*
