# Supplementary material for: Flowing between gongs: Mixed-methods insights into shared flow and temporal distortion in music performance
Source: PLoS One. 2025 Feb 10;20(2):e0302769. doi: 10.1371/journal.pone.0302769 (PMC11809782; doi:10.1371/journal.pone.0302769)
Supplement: S1 Table — Models are reduced in random effects in a stepwise manner while retaining all fixed effects. (DOCX) [file pone.0302769.s002.docx]

**S1 Table. Model comparisons of random effects.** Models are reduced in random effects in a stepwise manner while retaining all fixed effects.

|  | **time_distort**  All possible fixed and random | | | **time_distort**  Restrict correlations and intercepts | | | **time_distort**  Remove instrument slope | | |
| --- | --- | --- | --- | --- | --- | --- | --- | --- | --- |
| *Predictors* | *Estimates* | *CI* | *p* | *Estimates* | *CI* | *p* | *Estimates* | *CI* | *p* |
| (Intercept) | 0.08 | -0.86 – 1.02 | 0.857 | 0.19 | -0.80 – 1.19 | 0.696 | 0.13 | -0.86 – 1.12 | 0.793 |
| IF interact | -0.19 | -0.38 – 0.00 | 0.054 | -0.18 | -0.37 – 0.01 | 0.067 | -0.18 | -0.38 – 0.01 | 0.064 |
| condition (notation vs without) | -0.03 | -0.46 – 0.41 | 0.869 | -0.02 | -0.47 – 0.43 | 0.899 | -0.02 | -0.44 – 0.41 | 0.908 |
| condition (memorised vs improvised) | -0.10 | -0.85 – 0.66 | 0.714 | -0.09 | -0.82 – 0.65 | 0.733 | -0.08 | -0.76 – 0.60 | 0.739 |
| IF absorb | 0.20 | 0.02 – 0.37 | **0.029** | 0.20 | 0.02 – 0.38 | **0.027** | 0.20 | 0.02 – 0.38 | **0.026** |
| Instrument (structural vs elaborating) | -0.06 | -0.55 – 0.43 | 0.685 | -0.08 | -0.48 – 0.32 | 0.568 | -0.15 | -0.35 – 0.05 | 0.145 |
| Instrument (balungan vs solo) | 0.01 | -0.21 – 0.23 | 0.917 | 0.02 | -0.18 – 0.22 | 0.809 | 0.03 | -0.16 – 0.22 | 0.746 |
| pre flow | -0.01 | -0.14 – 0.11 | 0.819 | -0.03 | -0.16 – 0.11 | 0.661 | -0.02 | -0.16 – 0.11 | 0.740 |
| post flow | -0.00 | -0.15 – 0.14 | 0.956 | 0.01 | -0.14 – 0.16 | 0.928 | -0.01 | -0.15 – 0.14 | 0.944 |
| music training | -0.10 | -0.54 – 0.34 | 0.530 | -0.08 | -0.51 – 0.34 | 0.569 | -0.08 | -0.51 – 0.34 | 0.577 |
| IF interact X condition (notation vs without) | 0.06 | -0.33 – 0.45 | 0.754 | 0.02 | -0.37 – 0.41 | 0.925 | 0.00 | -0.40 – 0.40 | 0.999 |
| IF interact X condition (memorised vs improvised) | -0.07 | -0.54 – 0.40 | 0.765 | -0.07 | -0.54 – 0.41 | 0.777 | -0.04 | -0.52 – 0.44 | 0.862 |
| IF absorb X condition (notation vs without) | 0.09 | -0.22 – 0.41 | 0.554 | 0.13 | -0.19 – 0.45 | 0.425 | 0.13 | -0.20 – 0.45 | 0.438 |
| IF absorb X condition (memorised vs improvised) | 0.04 | -0.37 – 0.45 | 0.840 | 0.05 | -0.36 – 0.46 | 0.808 | 0.04 | -0.38 – 0.46 | 0.845 |
| **Random Effects** | | | | | | | | | |
| σ^2^ | 0.08 | | | 0.08 | | | 0.09 | | |
| τ_00_ | 0.02 _id_n.group_ | | | 0.02 _id_n.group_ | | | 0.02 _id_n.group_ | | |
|  | 0.00 _group_ | | | 0.00 _group_ | | | 0.00 _group_ | | |
|  | 0.04 _group.1_ | | |  | | |  | | |
|  | 0.01 _group.2_ | | |  | | |  | | |
|  | 0.00 _group.3_ | | |  | | |  | | |
| τ_11_ | 0.06 _group.1.music_training_ | | | 0.05 _group.music_training_ | | | 0.06 _group.music_training_ | | |
|  | 0.02 _group.2.instrument struct_vs_elab_ | | | 0.06 _group.instrumentStructural_ | | | 0.19 _group.cont_condNotated_ | | |
|  | 0.00 _group.2.instrument bal_vs_solo_ | | | 0.01 _group.instrumentBalungan_ | | | 0.14 _group.cont_condMemorised_ | | |
|  | 0.06 _group.3. cond notasi_vs_without_ | | | 0.02 _group.instrumentSolo_ | | | 0.03 _group.cont_condImprov_ | | |
|  | 0.19 _group.3. cond mem_vs_improv_ | | | 0.13 _group.cont_condNotated_ | | |  | | |
|  |  | | | 0.11 _group.cont_condMemorised_ | | |  | | |
|  |  | | | 0.02 _group.cont_condImprov_ | | |  | | |
| ρ_01_ | -1.00 _group.1_ | | |  | | |  | | |
|  | 1.00 _group.2.instrumentstructure_vs_elab_ | | |  | | |  | | |
|  | -1.00 _group.2.instrumentbalungan_vs_solo_ | | |  | | |  | | |
|  | -1.00 _group.3.cont_condnotasi_vs_without_ | | |  | | |  | | |
|  | -1.00 _group.3.cont_condmem_vs_improv_ | | |  | | |  | | |
| N | 4 _group_ | | | 4 _group_ | | | 4 _group_ | | |
|  | 33 _id_n_ | | | 33 _id_n_ | | | 33 _id_n_ | | |
| Observations | 99 | | | 99 | | | 99 | | |
| Marginal R^2^ / Conditional R^2^ | 0.176 / NA | | | 0.180 / NA | | | 0.173 / NA | | |
| AIC | 167.367 | | | 166.513 | | | 155.291 | | |

**S1 Table. (continued) Model comparisons of random effects.** Models are reduced in random effects in a stepwise manner while retaining all fixed effects.

|  | **time_distort**  Remove group intercept | | | **time_distort**  Remove musical training slope | | | **time_distort**  Remove condition slope | | |
| --- | --- | --- | --- | --- | --- | --- | --- | --- | --- |
| *Predictors* | *Estimates* | *CI* | *p* | *Estimates* | *CI* | *p* | *Estimates* | *CI* | *p* |
| (Intercept) | 0.13 | -0.86 – 1.12 | 0.793 | -0.06 | -1.18 – 1.06 | 0.917 | 0.21 | -1.04 – 1.47 | 0.733 |
| IF interact | -0.18 | -0.38 – 0.01 | 0.064 | -0.18 | -0.38 – 0.02 | 0.077 | -0.26 | -0.49 – -0.03 | **0.027** |
| condition (notation vs without) | -0.02 | -0.44 – 0.41 | 0.908 | -0.03 | -0.45 – 0.39 | 0.859 | -0.07 | -0.26 – 0.11 | 0.420 |
| condition (memorised vs improvised) | -0.08 | -0.76 – 0.60 | 0.739 | -0.09 | -0.75 – 0.58 | 0.714 | -0.10 | -0.32 – 0.13 | 0.395 |
| IF absorb | 0.20 | 0.02 – 0.38 | **0.026** | 0.18 | -0.01 – 0.37 | 0.059 | 0.22 | -0.00 – 0.44 | 0.053 |
| Instrument (structural vs elaborating) | -0.15 | -0.35 – 0.05 | 0.145 | -0.16 | -0.38 – 0.05 | 0.139 | -0.25 | -0.48 – -0.01 | **0.042** |
| Instrument (balungan vs solo) | 0.03 | -0.16 – 0.22 | 0.746 | 0.06 | -0.15 – 0.27 | 0.573 | 0.07 | -0.18 – 0.31 | 0.593 |
| pre flow | -0.02 | -0.16 – 0.11 | 0.740 | 0.01 | -0.14 – 0.16 | 0.931 | -0.04 | -0.21 – 0.14 | 0.684 |
| post flow | -0.01 | -0.15 – 0.14 | 0.944 | -0.08 | -0.25 – 0.09 | 0.375 | -0.11 | -0.31 – 0.09 | 0.283 |
| music training | -0.08 | -0.51 – 0.34 | 0.577 | -0.14 | -0.28 – -0.01 | **0.042** | -0.21 | -0.37 – -0.05 | **0.011** |
| IF interact X condition (notation vs without) | 0.00 | -0.40 – 0.40 | 0.999 | 0.03 | -0.38 – 0.45 | 0.871 | 0.33 | -0.13 – 0.79 | 0.154 |
| IF interact X condition (memorised vs improvised) | -0.04 | -0.52 – 0.44 | 0.862 | -0.08 | -0.58 – 0.42 | 0.745 | -0.29 | -0.85 – 0.28 | 0.314 |
| IF absorb X condition (notation vs without) | 0.13 | -0.20 – 0.45 | 0.438 | 0.11 | -0.23 – 0.44 | 0.529 | -0.06 | -0.44 – 0.32 | 0.741 |
| IF absorb X condition (memorised vs improvised) | 0.04 | -0.38 – 0.46 | 0.845 | 0.07 | -0.37 – 0.50 | 0.761 | 0.24 | -0.25 – 0.72 | 0.338 |
| **Random Effects** | | | | | | | | | |
| σ^2^ | 0.09 | | | 0.09 | | | 0.12 | | |
| τ_00_ | 0.02 _id_n.group_ | | | 0.05 _id_n:group_ | | | 0.08 _id_n:group_ | | |
| τ_11_ | 0.06 _group.music_training_ | | | 0.16 _group1.cont_condNotated_ | | |  | | |
|  | 0.19 _group.cont_condNotated_ | | | 0.12 _group2.cont_condMemorised_ | | |  | | |
|  | 0.14 _group.cont_condMemorised_ | | | 0.03 _group3.cont_condImprov_ | | |  | | |
|  | 0.03 _group.cont_condImprov_ | | |  | | |  | | |
| ρ_01_ |  | | |  | | |  | | |
| ρ_01_ |  | | |  | | |  | | |
| ICC |  | | | 0.34 | | | 0.41 | | |
| N | 4 _group_ | | | 4 _group_ | | | 33 _id_n_ | | |
|  | 33 _id_n_ | | | 33 _id_n_ | | | 4 _group_ | | |
| Observations | 99 | | | 99 | | | 99 | | |
| Marginal R^2^ / Conditional R^2^ | 0.173 / NA | | | 0.145 / 0.438 | | | 0.235 / 0.548 | | |
| AIC | 153.291 | | | 157.481 | | | 165.997 | | |
