# Supplementary material for: Flowing between gongs: Mixed-methods insights into shared flow and temporal distortion in music performance
Source: PLoS One. 2025 Feb 10;20(2):e0302769. doi: 10.1371/journal.pone.0302769 (PMC11809782; doi:10.1371/journal.pone.0302769)
Supplement: S2 Table — The most optimal model of S1 Table is reduced in fixed effects by means of AIC criterion improvement. (DOCX) [file pone.0302769.s003.docx]

**S2 Table. Model comparisons of fixed effects.** The most optimal model of S1 table is reduced in fixed effects by means of AIC criterion improvement.

|  | **time_distort**  Refit with maximum likelihood estimation | | | **time_distort**  Remove pre-flow | | | **time_distort**  Remove interaction with IF interaction | | | **time_distort**  Remove interaction with IF absorption instead | | |
| --- | --- | --- | --- | --- | --- | --- | --- | --- | --- | --- | --- | --- |
| *Predictors* | *Estimates* | *CI* | *p* | *Estimates* | *CI* | *p* | *Estimates* | *CI* | *p* | *Estimates* | *CI* | *p* |
| (Intercept) | 0.22 | -0.93 – 1.36 | 0.703 | -0.04 | -0.16 – 0.08 | 0.527 | -0.03 | -0.15 – 0.09 | 0.610 | -0.03 | -0.15 – 0.10 | 0.671 |
| IF interact | -0.26 | -0.48 – -0.05 | **0.018** | -0.27 | -0.48 – -0.05 | **0.016** | -0.31 | -0.49 – -0.12 | **0.001** | -0.32 | -0.51 – -0.13 | **0.001** |
| condition (notation vs without) | -0.07 | -0.24 – 0.10 | 0.394 | -0.08 | -0.24 – 0.09 | 0.371 | -0.02 | -0.18 – 0.14 | 0.802 | -0.06 | -0.22 – 0.11 | 0.489 |
| condition (memorised vs improvised) | -0.10 | -0.30 – 0.11 | 0.360 | -0.10 | -0.30 – 0.11 | 0.359 | -0.15 | -0.35 – 0.05 | 0.138 | -0.11 | -0.32 – 0.09 | 0.272 |
| IF absorb | 0.22 | 0.01 – 0.42 | **0.039** | 0.22 | 0.02 – 0.43 | **0.029** | 0.26 | 0.08 – 0.45 | **0.006** | 0.27 | 0.09 – 0.45 | **0.004** |
| Instrument (structural vs elaborating) | -0.25 | -0.47 – -0.03 | **0.028** | -0.25 | -0.47 – -0.03 | **0.027** | -0.25 | -0.47 – -0.03 | **0.029** | -0.25 | -0.47 – -0.03 | **0.029** |
| Instrument (balungan vs solo) | 0.07 | -0.16 – 0.29 | 0.564 | 0.08 | -0.14 – 0.30 | 0.471 | 0.06 | -0.16 – 0.28 | 0.590 | 0.08 | -0.14 – 0.29 | 0.479 |
| pre flow | -0.04 | -0.19 – 0.12 | 0.651 |  |  |  |  |  |  |  |  |  |
| post flow | -0.11 | -0.29 – 0.08 | 0.247 | -0.14 | -0.27 – -0.01 | **0.041** | -0.12 | -0.25 – 0.00 | 0.058 | -0.13 | -0.26 – -0.00 | **0.042** |
| music training | -0.21 | -0.36 – -0.07 | **0.005** | -0.22 | -0.36 – -0.08 | **0.004** | -0.22 | -0.36 – -0.07 | **0.004** | -0.22 | -0.36 – -0.07 | **0.004** |
| IF interact X  condition (notation vs without) | 0.33 | -0.09 – 0.76 | 0.125 | 0.33 | -0.10 – 0.76 | 0.127 |  |  |  | 0.26 | 0.06 – 0.46 | **0.012** |
| IF interact X  condition (memorised vs improvised) | -0.28 | -0.81 – 0.24 | 0.289 | -0.29 | -0.82 – 0.23 | 0.268 |  |  |  | -0.04 | -0.28 – 0.19 | 0.715 |
| IF absorb X  condition (notation vs without) | -0.06 | -0.42 – 0.29 | 0.725 | -0.06 | -0.41 – 0.29 | 0.742 | 0.18 | 0.02 – 0.35 | **0.033** |  |  |  |
| IF absorb X  condition (memorised vs improvised) | 0.23 | -0.22 – 0.68 | 0.308 | 0.24 | -0.21 – 0.69 | 0.292 | 0.03 | -0.18 – 0.24 | 0.797 |  |  |  |
| **Random Effects** | | | | | | | | | | | | |
| σ^2^ | 0.10 | | | 0.10 | | | 0.11 | | | 0.11 | | |
| τ_00_ | 0.07 _id_n:group_ | | | 0.07 _id_n:group_ | | | 0.07 _id_n:group_ | | | 0.07 _id_n:group_ | | |
| ICC | 0.39 | | | 0.40 | | | 0.38 | | | 0.38 | | |
| N | 33 _id_n_ | | | 33 _id_n_ | | | 33 _id_n_ | | | 33 _id_n_ | | |
|  | 4 _group_ | | | 4 _group_ | | | 4 _group_ | | | 4 _group_ | | |
| Observations | 99 | | | 99 | | | 99 | | | 99 | | |
| Marginal R^2^ / Conditional R^2^ | 0.266 / 0.554 | | | 0.258 / 0.553 | | | 0.246 / 0.533 | | | 0.254 / 0.541 | | |
| AIC | 166.015 | | | 161.087 | | | 157.395 | | | 155.470 | | |
