# Supplementary material for: Flowing between gongs: Mixed-methods insights into shared flow and temporal distortion in music performance
Source: PLoS One. 2025 Feb 10;20(2):e0302769. doi: 10.1371/journal.pone.0302769 (PMC11809782; doi:10.1371/journal.pone.0302769)
Supplement: Attachment — Submitted filename: PLOS.docx [file pone.0302769.s004.docx]

This research is very interesting, especially in trying to find the flow of gamelan players. Firstly, for understanding the qualitative data, there should be an exploration of cultural aspects because gamelan is not music in the same sense as understood by the Greeks. Almost all musical elements present in Western culture are different from gamelan, which does not have the concepts of intervals, melody, chord progression, harmony, improvisation, etc. The flow/durée in gamelan playing is very close to the everyday life of the Javanese, who are conflict-averse, along with the addition of playing experienced.

Secondly, Javanese gamelan is the sound behavior of the Javanese, more as a representation of conversation and cultural relations in a non-verbal way. In a gamelan group, all ways of life of the Javanese people can be found, and they usually place great importance on “rasa” (feeling). The essence of gamelan playing, whether achieving flow or peak experience, is entirely based on and considered according to “rasa,” which is also mentioned in Indian music.

Thirdly, for the quantitative aspect with repeated measures, it should be clarified further because the self-report used is different from the measurement scale.
